# Hepatoid adenocarcinoma arising from heterotopic pancreas of the ileum: A case report

**DOI:** 10.1097/MD.0000000000004067

**Published:** 2016-08-19

**Authors:** Ling Tong, Huaxiong Pan, Jun He, Mixia Weng, Liduan Zheng

**Affiliations:** a Department of Pathology, Chifeng Municipal Hospital, Chifeng, Inner Mongolia Autonomous Region; b Department of Pathology, Union Hospital of Tongji Medical College, Huazhong University of Science and Technology, Wuhan, China.

**Keywords:** α-fetoprotein, hepatoid adenocarcinoma, heterotopic pancreas, ileum

## Abstract

**Introduction:**

Hepatoid adenocarcinoma (HAC) is a rare neoplasm with a striking morphologic similarity to hepatocellular carcinoma. The most common sites of HAC are the stomach, lung, and pancreas.

**Case report:**

Here we report a rare case of HAC arising from the heterotopic pancreas (Heinrich type II) in the ileum with lymph node metastasis. A 56-year-old man was admitted to our hospital presenting with bloody stools under no obvious predisposing causes. The colonoscopy and the gastroscopy showed no pathological findings. A computed tomography scan showed an intussusception of ileum. Then partial resection of ileum was performed with end-to-end anastomosis and appendectomy. Histopathological examination showed a malignant transformation of heterotopic pancreas (Heinrich type II) in the ileum. We made the diagnosis of HAC based on clinical pathological features and immunochemical staining. The patient received chemotherapy and died 9 months later.

**Conclusion:**

To our best knowledge, this is the first reported case of HAC originated from a heterotopic pancreas in the ileum. The clinical pathological features and immunochemical staining are important for correct diagnosis of HAC.

## 1 Introduction

Hepatoid adenocarcinoma (HAC) is a rare neoplasm with a striking morphologic similarity to hepatocellular carcinoma. The most common sites of HAC are the stomach, lung, and pancreas. Heterotopic pancreas is an uncommon gastrointestinal malformation. The common site is in the antrum of stomach, because the chance of endoscopic observation and organ resection is much more frequent than in other organs. Several other sites reported in the literatures include duodenum, stomach, jejunum, ileum, Meckel diverticulum, and gallbladder.^[1–8]^ Heterotopic pancreas was first described in 1909 and has been classified into 3 types.^[9]^ Type I consists of typical pancreatic tissues with all pancreatic cell types present—acini, ducts, and islet cells. Type II is composed of pancreatic tissues with ducts and acini. Islet cells are absent. Type III is composed of pancreatic tissue with few acini and large numbers of ducts, some of which are cystically dilated.

The majority of heterotopic pancreatic nodules are small (<1 cm) and appear to be asymptomatic. However, larger nodules can have nonspecific symptoms including abdominal pain, melaena, anaemia, intussusception, obstruction, and cystic change.^[10–12]^ Heterotopic pancreas in ileum often causes intussusception.^[4,5]^ Several rare cases of carcinomas arising within heterotopic pancreatic tissue have been reported.^[1,3,11,13–17]^ However, HAC arising in ileal heterotopic pancreas has not been described previously. Here we report a rare case of HAC arising from the heterotopic pancreas (Heinrich type II) in the ileum with lymph node metastasis.

## 2 Case report

**Figure 1:**
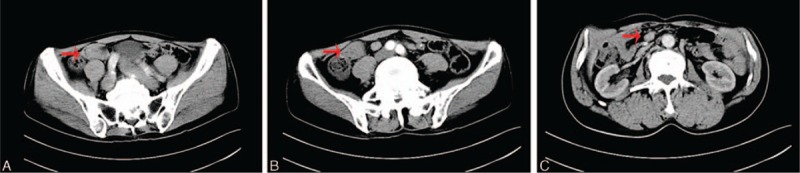
Abdominal CT scan of HAC arising from heterotopic pancreas in the ileum. A, CT scan showed an equal density nodule sized about 2.5 cm × 1.8 cm located on the proximal ileum (indicated by red arrow). B, We found no obvious enhancement in the contrast (indicated by red arrow). C, The edge of the ileum was smooth and clear, and we found no malignant sign, but 1 enlarged lymph node (about 1.7 cm × 1.1 cm) on the mensentery (indicated by red arrow). CT = computed tomography, HAC = hepatoid adenocarcinoma.

A 56-year-old male patient presented with bloody stools with no obvious predisposing causes, 5 times a day, 300 mL every time. He visited our hospital. The main laboratory findings were normal. Colonoscopy and gastroscopy showed no pathological findings. A computed tomography scan showed an intussusception of ileum and intrahepatic bile duct stones with mild bile duct expansion (Fig. 1). Other abdominal organs showed no abnormal findings.

The serum carcinoembryonic antigen level was elevated (15.6 μg/L, reference <5.0 μg/L), whereas serum levels of α-fetoprotein (AFP) (2.6 μg/L, reference <13.4 μg/L) and CA19–9 (9.1 U/mL, reference <37 U/mL) were normal. The patient underwent partial resection of ileum with end-to-end anastomosis and appendectomy. Then he received chemotherapy in a local hospital; the regimen was unknown. He died 9 months after the surgery.

Small intestinal intussusception was 10 cm long, 80 cm from the ileocecal valve, and a solid and gray white mass about 3cm × 2cm × 2 cm was found in the small intestine, which infiltrated the bowel wall to the deep muscularis propria. An enlarged lymph node was found in the mesentery.

**Figure 2:**
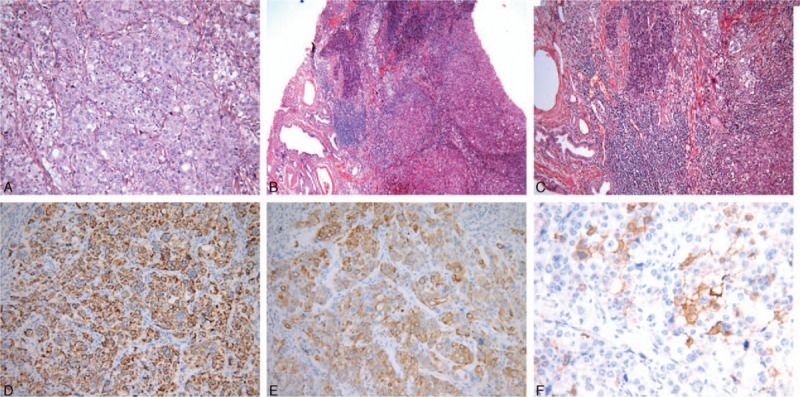
Histological and immunohistochemical analysis of HAC arising from the heterotopic pancreas in the ileum. A, The solid nests and trabecular structure mimicked the pattern of hepatocellular carcinoma of polygonal atypical cells, with eosinophilic cytoplasm and round-to-oval nuclei. B and C, At 1 edge of the tumor, pancreatic tissues were clearly defined with ducts and acini, and islet cells were absent, diagnosed as Heinrich type II heterotopic pancreas. There was merging of heterotopic pancreas with HAC. D, HepPar1-diffuse cytoplasmic staining of the tumor cells. E, CK7-diffuse cytoplasmic and membranous staining of the tumor cells. F, Villin-focal cytoplasmic staining of the tumor cells. A, D–F = magnification ×200, B = magnification ×40, C = magnification ×100. CK7 = cytokeratin 7, HAC = hepatoid adenocarcinoma, HepPar1 = hepatocyte paraffin 1.

Histology examination based on hematoxylin and eosin (HE) staining revealed that the tumor infiltrated the bowel wall to the deep muscularis propria, with striking morphologic similarity to hepatocellular carcinoma. The tumor cells arranged in an organoid pattern with solid nests, and trabecular structures of large-to-medium cells with eosinophilic cytoplasm and round-to-oval nuclei (Fig. 2A). Associated with the tumor and at its periphery were pancreatic tissues with ducts and acini; islet cells were absent, diagnosed as Heinrich type II heterotopic pancreas (Fig. 2B and C). Residual heterotopic pancreatic tissue was detected just from the submucosal layer to the proper muscular layer. The only lymph node was positive with adenocarcinoma metastasis.

Immunohistochemical staining revealed that the tumor cells were strongly positive for hepatocyte paraffin 1 (HepPar1) and cytokeratin (CK)7, positive for Villin (Fig. 2D–F), but were negative for AFP, glypican-3, CK20, caudal-related homeobox 2 (CDX2), thyroid transcription factor-1 (TTF1), paired box 2 (PAX2), synaptophysin (Syn), and chromogranin A (CgA). Based on the clinicopathologic and immunohistochemical results, the final diagnosis was poorly differentiated HAC with lymph node involvement (pT3N1).

The written informed consent for this case report was obtained from the patient; no autopsy was performed and the consent procedure was approved by the Ethics Committee of Union Hospital.

## 3 Discussion

To the best of our knowledge, a case of HAC arising in ileal heterotopic pancreas has not been reported previously. Three criteria have been suggested for the diagnosis of adenocarcinoma arising from heterotopic pancreas. First, the tumor must be within or near the heterotopic pancreas; second, a transition between the pancreatic tissue and the tumor should be established; and third, the non-neoplastic pancreatic tissue should show well-developed ducts and acini.^[18]^ In the case we described here, all 3 criteria were met. Our case illustrates a HAC arising within the context of heterotopic pancreas in the ileum. Furthermore, the HAC appeared to merge with the heterotopic pancreatic tissue. However, the independent existence of 2 distinct pathologies—heterotopic pancreas and HAC—can not be entirely excluded.

Hepatoid adenocarcinoma is a rare but important neoplasm with a striking morphologic similarity to hepatocellular carcinoma. HAC exhibits specific hepatoid structures, such as solid/trabecular, pseudoglandular pattern and hyaline globules, and large polygonal eosinophilic cells with focal-associated tubular/papillary differentiated areas. HAC has been described in different organs such as the stomach,^[19]^ colon,^[20]^ jejunum,^[21]^ gallbladder,^[22,23]^ lung,^[24]^ and ovary.^[25]^ The stomach is the most common origin of the tumor. This neoplasm frequently presents at an advanced stage and is characterized in most cases by increased serum level of AFP, which is generally considered important for the diagnosis. AFP overproduction happens in most but not all the cases, thus it is important to note that AFP positivity is not necessarily diagnostic of HAC. Therefore, the diagnosis of HAC should be based on histological features of the tumor. In this case, serum AFP level was normal, whereas serum carcinoembryonic antigen level was elevated. In addition, AFP immunostaining was negative.

Immunohistochemically, many liver-specific proteins, including AFP, glypican-3, HepPar1, albumin, transferin, and alpha-1-antitrypsin, have been detected in the cytoplasm of HAC cells.^[24–26]^ Liu et al reported a case of HAC from the jejunum arising in ulcerative colitis, in which the hepatoid components were immunohistochemically positive for CK8, CK18, and CK19, AFP, HepPar1, and polyclonal carcinoembryonic antigen (pCEA; canalicular pattern), and negative for CK7 and CK20.^[21]^ In this case, a strong positivity with hepatocyte suggested the presence of tumors with hepatoid differentiation. The immunostaining pattern was focally granular in the cytoplasm of neoplastic cells. Hepatocyte antigen is recognized by antibody HepPar1, a widely used diagnostic marker for hepatocellular carcinoma and tumors with hepatoid differentiation. In addition, being positive for keratin 7 suggested the presence of an adenocarcinoma.

Hepatoid adenocarcinoma has a poor prognosis; a high portion of patients have metastasis at the time of diagnosis. The median survival of HAC patients is 12 months. Nearly half of the patients die within the first 12 months. In this case, although the patient underwent partial resection of ileum with end-to-end anastomosis and appendectomy followed by chemotherapy, he died 9 months after the surgery. One shortcoming related to our case is that we did not perform extensive follow-up.

In conclusion, HAC is a rare and highly aggressive malignancy with poor prognosis. The etiology of HAC is unknown. We present the first case of hepatoid adenocarcinoma arising from heterotopic pancreas in the ileum with lymph node metastasis. We made the diagnosis based on the combination of clinical pathological features and immunochemical staining.
